# Treatment of Angiomyoblastoma in a Young Lady in Saudi Arabia: A Case Report: Angiomyoblastoma in a Young pLady in Saudi Arabia

**DOI:** 10.31661/gmj.v14i.3861

**Published:** 2025-10-28

**Authors:** Roua Shoub Gbril Ali, Ghadeer Adel Mosfer Alghamdi, Raad Hadi Madkhali, Abdulaziz Saleh Alobaid, Zarqa Saleem

**Affiliations:** ^1^ Department of OBGYN, Care Medical Alrawabi, Riyadh; ^2^ Department of Radiology, Care Medical Alrawabi, Riyadh; ^3^ Dr Sulaiman Habib Olya, Riyadh

**Keywords:** Angiomyofibroblastoma, Vagina, Treatment, Surgical Excision, Young Lady

## Abstract

**Background:**

Background: Angiomyofibroblastoma (AMFB) is a rare, benign soft tissue tumor that belongs to the mesenchymal tumor category and affects the female genital tract. Aggressive angiomyxoma, a distinct stroma myxoedematous mesenchymal tumor with a significant risk of local recurrences, must be histomorphologically distinguished from AMFB. Radiography and histopathology are used for accurate diagnosis. The treatment depended on the complete surgical excision.

**Case Presentation:**

Case Presentation: Herein, we presented a case of a 36-year-old female (P3+0) presented with a left vaginal mass noted two years prior. The radiology report suggested angiomyxoma or myxoid liposarcoma as possible differentials. Surgical resection revealed a benign genital stromal tumor, specifically angiomyofibroblastoma (AMFB). After complete surgical excision, the immunohistochemical analysis supported the diagnosis of angiomyofibroblastoma. Two months postoperatively, an MRI Post-resection images show complete resection with no feasible scar, residual tumour, or recurrence.

**Conclusion:**

Conclusion: Our case is rare AMFB. AMFB may be missed when diagnosed with other masses, such as angiomyxoma or myxoid liposarcoma, so we need to do the necessary tests to diagnose and differentiate AMFB accurately from other masses. Ultrasound and MRI are initial diagnostic tools, while histopathology ensures the correct diagnosis. Surgical removal with free margins remains the appropriate treatment. Long-term follow-up is essential to monitor for potential recurrence, though AMFB typically carries an excellent prognosis.

## Introduction

Angiomyofibroblastoma (AMFB) is a rare, benign soft tissue tumor belonging to the mesenchymal tumor; it originates from blood vessels and stromal cells [1].

The woman’s genital tract, particularly the vagina and vulva, is where AMFB typically begins. The broad ligament, fallopian tube, and, less commonly, the male genital tract have also been identified as potential locations [2]. The highest prevalence rate was reported among young to middle-aged females [3].

Clinically, AFMB manifests as a slowly expanding mass; it lacks any other distinctive symptoms that set it apart from other tumors of the female genital tract [4]. Aggressive angiomyxoma, a distinct stroma myxoedematous mesenchymal tumor with a significant risk of local recurrences, must be histomorphologically distinguished from AMFB [5]. The gold standard of care is surgical removal [6]. Here, we present a case of a 36-year-old female diagnosed with vaginal AMFB, showing the diagnostic difficulties when considering this rare entity and available treatment.

## Case Presentation

**Figure-1:**
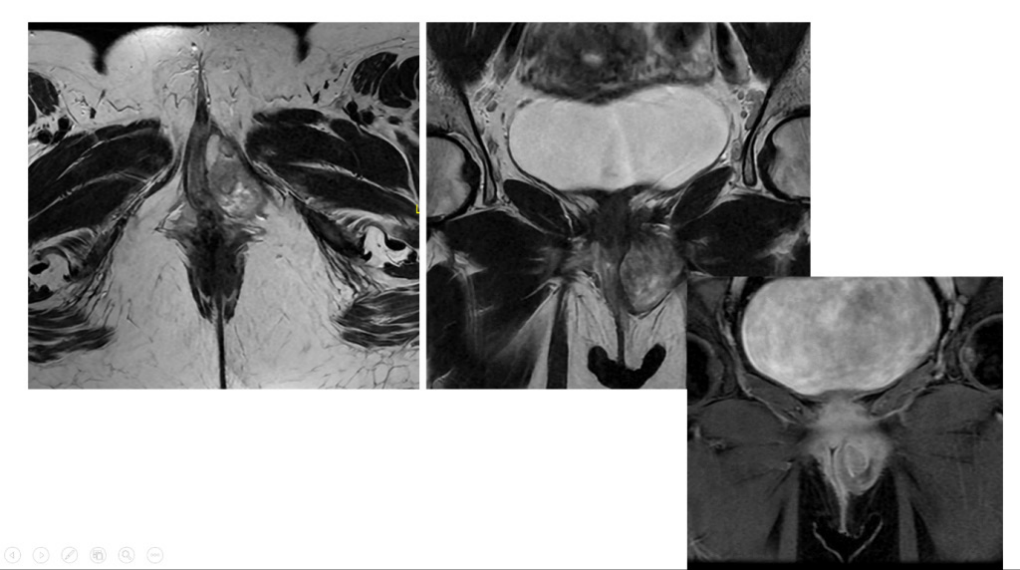
Figure 1. Preoperative MRI showed left pelvic mass measuring 5 x 3 x 4 cm. Show bright T2 signal intensity with a swirling appearance crossing the left levator ani muscle towards the left external anal sphincter abutting the sphincter.

**Figure-2:**
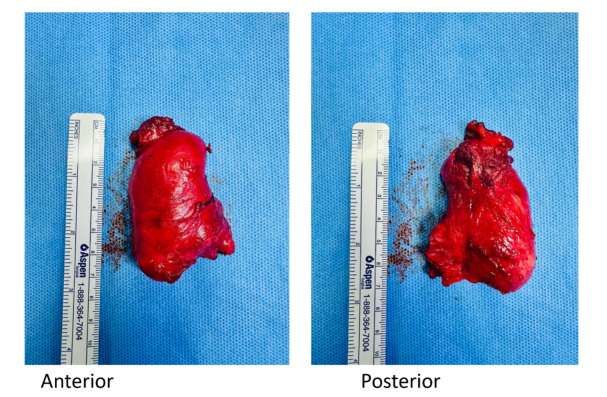
Figure 2. The anterior and posterior surfaces of a 6 cm tumor near the urethra and puborectalis muscle.

**Figure-3:**
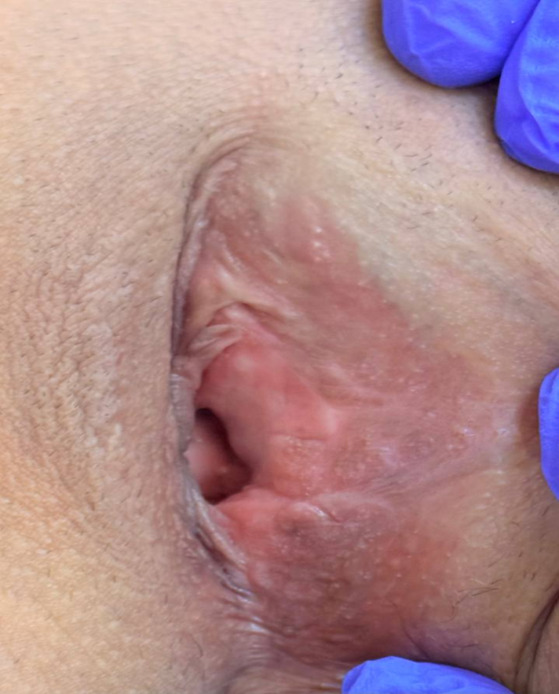
Figure 3. The postoperative site was closed using a parallel technique, ensuring optimal healing.

**Figure-4:**
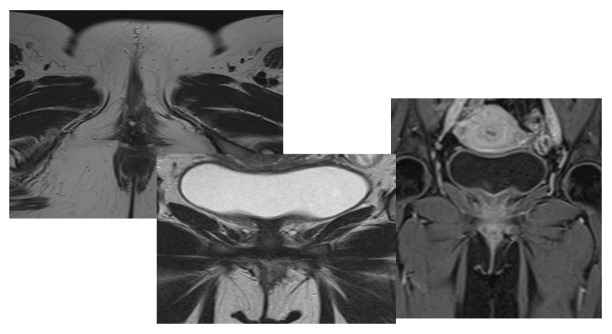
Figure 4. Post-contrast shows no significant abnormal enhancement.

A 36-year-old female (P3+0) presented with a left vaginal mass noted two years prior, which had gradually increased in size. She had a history of a vulvar mass first reported in November 2023 but no significant medical or surgical history.

The initial magnetic resonance imaging (MRI) from an external facility revealed a well-defined left lateral lower vaginal lesion extending to the perineum. The radiology report suggested angiomyxoma or myxoid liposarcoma as possible differentials. The patient underwent surgical removal of the mass in July 2024 at another hospital. However, no prior imaging or clinical history was available at that time.

Histopathology from the first surgery suggested aggressive angiomyxoma based on the spindle cell tumor’s morphology. However, the diagnosis was inconclusive due to incomplete excision. An MRI in August 2024 revealed Left pelvic mass measuring 5 x 3 x 4 cm. Show bright T2 signal intensity with a swirling appearance crossing the left levator ani muscle towards the left external anal sphincter abutting the sphincter. Internal sphincter and intersphincteric fat are intact. Mass extends to the left vaginal wall with an indentation of the vaginal wall at the level of the urethra. The mass shows avid enhancement post-contrast (Figure-1).

A second surgery was planned to completely excision the mass. The 6 cm tumor, located near the urethra and puborectalis muscle, was excised with meticulous dissection (Figure-2). The postoperative site was closed using a parallel technique, ensuring optimal healing (Figure-3).

Histopathology of the second excised mass revealed a well-encapsulated stromal tumor with spindle cells embedded in a myxoid stroma. Immunohistochemical analysis supported the diagnosis of angiomyofibroblastoma. Margins were free of tumor, confirming complete resection.

Two months postoperatively, an MRI Post-resection images show complete resection with no feasible scar, residual tumor, or recurrence. There is no vaginal wall deformity. Post-contrast shows no significant abnormal enhancement (Figure-4). Incision site healing was unremarkable, and the patient reported no new symptoms.

## Discussion

Fletcher et al. initially reported angiomyofibroblastoma (AMFB), a benign mesenchymal tumor, in 1992 [3]. Few instances of it occur in men, and it usually affects the external genital tract in women [7]. Women in their middle years (between the ages of 30 and 50) are generally affected by AMFB [3]. Our patient was a 36-year-old female.

Clinically, the tumor may be asymptomatic or manifest as a painless weight in the pelvis [8]. When it is found in the cervix, uterus, or urethral area, it can occasionally exhibit obstructive symptoms such as dysuria [9]. It is easily diagnosed since it is often seen in the superficial regions of the lower female genital tract. However, a tumor in the pelvis, peritoneal cavity, or ilia fossa may be discovered after it has grown considerably [10]. Regarding our case, she was symptomatic and admitted with a left vaginal mass noted two years prior, which had gradually increased in size.

Ultrasound (US) is the primary imaging technique used to evaluate vaginal masses due to its high resolution, availability, and cost-effectiveness. It can distinguish between vulvar and vaginal amyloid-beta (AMFB) and other mesenchymal tumors.[11]. AMFB is typically well-circumscribed on MRI and is often described as hypointense on T1W images, hyperintense on T2W images, and with homogenous hyperenhancement on Gd-C enhanced images. Contrast-enhanced imaging has been reported in five studies [11]. Regarding our case, the initial MRI suggested angiomyxoma or myxoid liposarcoma as possible differentials. After the first excision surgery, MRI shows bright T2 signal intensity with a swirling appearance crossing the left levator ani muscle towards the left external anal sphincter abutting the sphincter.

Malignant mesenchymal tumors, aggressive angiomyxoma, cellular angiofibroma, fibroepithelial stromal polyp, and superficial cervicovaginal myofibroblastoma are all included in the broad differential diagnosis. Usually more significant than 10 cm, aggressive angiomyxoma (AAM) is a locally infiltrative tumor of tiny, spindle- to stellate-shaped cells encased in a thick layer of a myxoid matrix with "pushing" infiltrative boundaries. Compared to angiomyofibroblastoma, the cells are often more evenly distributed and monomorphic [7]. AAM and AMFB have varied positivity for desmin and α-SMA but are immunohistochemically negative for S-100 and positive for vimentin, estrogen receptor, and progesterone receptor. On the other hand, a new molecular analysis confirms that AAM and AMFB are different types of cancer. A third of AAM patients had the HMGA2 gene arrangement reported, whereas AMFB cases do not [11]. Cellular angiofibroma, which has thicker blood arteries and lacks hormone receptor expression, is another differential diagnosis. Inflammatory myofibroblastic tumors and inflammatory fibroid polyps are further diseases that must be checked out [12][13].

Regarding our case, the radiology report suggested angiomyxoma or myxoid liposarcoma as possible differentials. Histopathology from the first surgery suggested aggressive angiomyxoma based on the spindle cell tumor’s morphology, but the diagnosis wasn’t accurate due to incomplete excision. A second surgery was planned to completely excision the mass. Histopathology of the second excised mass revealed a well-encapsulated stromal tumor with spindle cells embedded in a myxoid stroma. Immunohistochemical analysis supported the diagnosis of angiomyofibroblastoma. Margins were free of tumor, confirming complete resection.

Surgical removal is the gold standard treatment [6]. In the present case, Without any noticeable scarring, tumor recurrence, vaginal wall deformation, post-contrast enhancement, or new symptoms, the patient had a complete resection.

## Conclusion

Our case is rare AMFB. AMFB may be missed when diagnosed with other masses, such as angiomyxoma or myxoid liposarcoma, so we need to do the necessary tests to diagnose and differentiate AMFB accurately from other masses. Ultrasound and MRI are initial diagnostic tools, while histopathology ensures the correct diagnosis. Surgical removal with free margins remains the appropriate treatment. Long-term follow-up is nessesary to check for poten tial recurrence, though AMFB typically carries an excellent prognosis.

## Conflict of Interest

The authors declare that they have no conflict of interest.
